# Reaching national consensus on the core clinical skill outcomes for family medicine postgraduate training programmes in South Africa

**DOI:** 10.4102/phcfm.v9i1.1353

**Published:** 2017-05-26

**Authors:** Yusuf Akoojee, Robert Mash

**Affiliations:** 1 Division of Family Medicine and Primary Care, Stellenbosch University, South Africa

## Abstract

**Background:**

Family physicians play a significant role in the district health system and need to be equipped with a broad range of clinical skills in order to meet the needs and expectations of the communities they serve. A previous study in 2007 reached national consensus on the clinical skills that should be taught in postgraduate family medicine training prior to the introduction of the new speciality. Since then, family physicians have been trained, employed and have gained experience of working in the district health services. The national Education and Training Committee of the South African Academy of Family Physicians, therefore, requested a review of the national consensus on clinical skills for family medicine training.

**Methods:**

A Delphi technique was used to reach national consensus in a panel of 17 experts: family physicians responsible for training, experienced family physicians in practice and managers responsible for employing family physicians.

**Results:**

Consensus was reached on 242 skills from which the panel decided on 211 core skills, 28 elective skills and 3 skills to be deleted from the previous list. The panel was unable to reach consensus on 11 skills.

**Conclusion:**

The findings will guide training programmes on the skills to be addressed and ensure consistency across training programmes nationally. The consensus will also guide formative assessment as documented in the national portfolio of learning and summative assessment in the national exit examination. The consensus will be of interest to other countries in the region where training programmes in family medicine are developing.

## Introduction

International policy guidelines stipulate that registrars in family medicine should be taught all the procedures within their scope of practice during training. The minimum requirement would be to cover all procedures typically performed by a substantial number of practising family physicians (FPs) in both ambulatory and inpatient settings. Ideally, these procedures should be taught by FPs and the list should be updated on a regular basis to include new or emerging procedures.^1^ Unfortunately, there is not always adequate coverage of all procedures or consistency between training programmes, even in the same country. An agreed national procedure list may help to guide training programmes.^2^

The training of family medicine registrars in clinical skills is a crucial element in the development of FPs for district health services in South Africa.^3^ FPs who are competent in a comprehensive range of clinical skills should be able to strengthen district health services and improve patient health outcomes.^4^ Competency in these clinical skills should ensure that patients are able to access care locally, thereby avoiding referral or further progression of their disease, when it may become more serious and costly to treat.^5^

FPs in South Africa serve a wide spectrum of patients. The clinical skills required by FPs are determined by the burden of disease, the settings where FPs work, the resource constraints of the health care system and the policy on the package of services expected.^2^ FPs should be familiar with the health needs in the communities they serve and should be equipped with and competent in performing the relevant clinical skills in order to manage patients comprehensively, thereby improving patient care and patient health outcomes.^6^

FPs work in a number of different care settings, such as the private or public health care sectors, rural or urban settings and community health centres or district hospitals.^7^ FPs who work in district hospitals require more procedural clinical skills compared to those who have chosen a career in primary care.

These diverse practice settings emphasise the significant role FPs play in the district health care system and the broad scope of practice required. In South Africa, FPs often work in remote or underserved communities where resources are scarce, emergency medical services limited and referral hospitals distant and overloaded, making it difficult to transfer patients.^8^

Patients that should be managed at a higher level are therefore often managed in the district health services, which puts further pressure on the clinical skills expected of FPs. Conversely, large urban district hospitals may also employ FPs and expect a range of skills more appropriate to regional or tertiary hospitals. Clinical skills training is therefore vital for family medicine registrars to prepare them for their involvement in inpatient district hospital and ambulatory primary health care.^3^

Since the official introduction of family medicine registrar training in South Africa, there have been two previous publications on the clinical skills required. The first study ‘Building consensus on clinical procedural skills for South African family medicine training using the Delphi technique’ proposed a set of essential clinical skills for the training of FPs, which served as a benchmark for South African family medicine training programmes.^4^

The second article ‘Obtaining consensus on core clinical skills for training in family medicine’ presented the final consensus of the College of Family Physicians and the Academy of Family Physicians on the required skills based on the prior research study.^9^ The final skills list produced represented the consensus of family medicine educators in South Africa and provided a basis for family medicine registrar training.

The departments of family medicine accepted that the agreed list was a dynamic one that may need to be updated to reflect changing circumstances and lessons learnt and proposed that the list be reviewed.^9^ This study, therefore, intends to revise and update the list of clinical skills, as it is now almost 10 years since the original work was done.

Refining the clinical skills required will help training programmes to revise their learning outcomes and to ensure consistency across training programmes nationally. The skills list also guides formative assessment and learning as documented in the national portfolio of learning as well as summative assessment in the national exit examination offered by the College of Family Physicians. The skills list also guides the type of rotations and clinical training required for each registrar in each specific training complex. The list can also guide quality management and assurance of the training programmes and supervisors.

The family medicine training programmes coordinate their activities with the help of the National Education and Training Committee of the South African Academy of Family Physicians. This committee had specifically requested a review of the national consensus on clinical skills for family medicine training.

Postgraduate training in family medicine is also developing in many countries in sub-Saharan Africa, for example, Botswana, Zimbabwe, Kenya, Uganda, Nigeria and Ghana.^10^ This study will also be of interest to universities or colleges that are developing or revising curricula.

## Aim and objectives

The aim was to reach a new national consensus on the clinical skill outcomes for family medicine postgraduate training programmes in South Africa. Objectives were as follows:

- to reach consensus on the clinical skills that should be retained from the current list
- to reach consensus on the clinical skills to be omitted from the current list
- to reach consensus on the clinical skills to be added to the list
- to reach consensus on the clinical skills that may be seen as optional or elective skills.

## Methods

### Study design

The Delphi method was used to reach consensus on the clinical skills required amongst a group of experts from across the country. The key features of this design were anonymity of the panel members, iteration with controlled feedback from a series of questionnaires, quantitative analysis of the group’s response and the use of explicitly defined expert opinion.^11^

### Study setting

At the time of this study, there were eight postgraduate programmes in family medicine offered by the eight medical schools situated at the Universities of Cape Town, Stellenbosch, Free State, Witwatersrand, Pretoria, Limpopo, KwaZulu-Natal and Walter Sisulu. Training was organised in primary health care facilities and district and regional hospitals, with formal training posts and direct supervision by FPs or other specialists. Registrars rotated through the various facilities and clinical departments to attain the learning outcomes. The national portfolio of learning guided workplace-based training and assessment and included a logbook for assessment of competency in clinical skills.^12^ Registrars provided evidence in the portfolio of three years of full-time training in order to gain entrance to the national exit examination offered by the College of Family Physicians. The examination included assessment of clinical skills through written papers and an objective structured clinical examination and observation of consultation in three clinical cases.

Upon completion of training, FPs could apply for advertised FP posts in primary health care or district hospitals within the public sector or become general practitioners within the private sector. In the public sector, the role of the FP has been defined as a competent clinician able to work at the district hospital or in primary care, a consultant to the rest of the health care team, a capacity builder who strengthens the clinical skills of the whole team, a leader of clinical governance to improve the quality of care, a champion of community-orientated primary care and in some cases a supervisor or trainer of under- or postgraduate students.^13^

### Study population

A panel of 36 experts was identified and invited to serve on the Delphi panel:

- heads of family medicine departments (eight people).
- postgraduate programme managers (eight people).
- experienced FPs in practice, four in primary care and four in district hospitals (eight people).
- managers in the Department of Health at national, provincial or district levels (eight people).
- specialists from other disciplines for specific high procedure areas – surgery, anaesthetics, obstetrics and orthopaedics – who are involved in training registrars in family medicine or district clinical specialist teams (four people).

### Data collection

The Delphi technique uses an iterative strategy to gather information and arrive at consensus. The panel of experts were asked to respond to a series of questionnaires that were distributed to them electronically. It was anticipated by the research team that up to four rounds would be required to obtain consensus on all items.

The first questionnaire was based on the existing list of clinical skills, the current policy on the package of care required by district hospitals and primary care, as well as local research on skills required at district hospitals.

The participants were asked to select one of the following options for each clinical skill:

- retain skill – FPs must be competent to perform this skill
- delete skill – FPs do not need to perform this skill
- optional or elective skill – FPs may be required to perform this skill in certain limited settings such as rural or remote district hospitals
- space was provided for qualitative feedback on additional clinical skills that should be added or comments on the list of clinical skills.

The expert panel members were asked to respond to a series of three questionnaires, which were distributed to them electronically via email over a period of five months, from July to December 2015.

### Data analysis

Demographic and profile data were collected on the panel members. Consensus was defined as 70% or more of the group agreeing on an option for that specific skill. The analysis involved descriptive statistics in terms of numbers and frequencies. Once consensus was reached, an item was removed from the questionnaire in the next round. Feedback was provided to the group on the responses of the panel in the previous round and the qualitative feedback was used to revise the questionnaire.

### Ethical consideration

Ethical approval was obtained from the Stellenbosch Health Research Ethics Committee 1 via expedited review procedures with the protocol number (S14/07/157) on 15 October 2015.

## Results

From the panel of 36 experts who were invited, 17 agreed (three heads of family medicine departments, three postgraduate programme managers, six experienced FPs; three managers from the Department of Health at national, provincial or district levels and two specialists from high specific procedure areas). Out of these 17 panel members, nine were located in the Western Cape, three in Eastern Cape, three in Gauteng, one in KwaZulu-Natal and one in the Free State. Six of the eight training programmes at the universities were represented (Walter Sisulu, Witwatersrand, KwaZulu-Natal, Stellenbosch, Pretoria, Free State, but not Cape Town and Limpopo). Overall, 10 panel members were men and 14 respondents were aged between 45 and 64 years. Seventeen questionnaires were returned from each of the three rounds giving an overall response rate of 100% amongst those that consented.

### First questionnaire

The first questionnaire contained a list of 238 clinical skills and consensus was reached on 193 of the skills in the first round where the panel decided that 187 skills should be retained and 6 skills should be made elective. The panel could not reach consensus on 45 skills from the first round.

### Second questionnaire

All skills on which consensus had been reached in the first questionnaire (193 skills) were deleted from the second questionnaire. The results for the 45 skills on which there was no consensus from the first round were included in the second questionnaire and distributed to the panel members. In the second round, 17 skills were added from the qualitative feedback of the panel members in the first round. There were, therefore, a total of 62 skills in the second round. Consensus was reached on 42 clinical skills and the panel decided that 23 skills should be retained and 19 skills should be made elective. The panel could not reach consensus on 20 skills in the second round.

### Third questionnaire

In the final round, the remaining 20 skills were again presented to the panel with the results obtained in the second round as feedback to the panel. Consensus was reached on nine clinical skills and the panel decided that six should be elective skills and three clinical skills should be deleted. The panel could not reach consensus on 11 skills in the last round.

### Final consensus

The final consensus achieved for the clinical skills is presented in Tables 1–4.

**TABLE 1:** Core clinical skills for the training of family physicians (*N* = 17).

Consensus score (%)	Clinical skill
**Perform common side-room tests**	
88	Use a glucometer
88	Use a haemoglobinometer
88	Perform a pregnancy test
94	Perform urinalysis
94	Venepuncture
**Adult health – general**	
94	Femoral vein puncture
100	Lumbar puncture
88	Routine intravenous access in adults
88	Lymph node excision biopsy
77	Perform point-of-care testing for HIV
**Adults – musculoskeletal**	
82	Measure shortening of the legs
94	Aspirate and inject the knee joint
94	Inject tennis elbow or golfer’s elbow
100	Interpret radiographs of joints
82	Inject carpal tunnel syndrome
76	Inject De Quervain’s tenosynovitis
82	Inject the shoulder and subacromial bursa
77	Inject trochanteric bursitis
**Adults – abdomen**	
94	Test stool for occult blood
100	Incision and drainage of perianal haematoma
100	Interpret the abdominal radiograph in an adult
94	Proctoscopy
76	Interpret barium swallows
**Adults – chest**	
100	Electrocardiogram set up, record and interpret
100	Interpret chest radiograph
100	Measure peak expiratory flow
94	Nebulise a patient
100	Pleural tap
100	Use inhalers and spacers
88	Exercise stress test
70	Perform and interpret office spirometry
**Adults – urology**	
100	Penile block
100	Reduce a paraphimosis
100	Circumcision
100	Drain hydrocele
94	Insert a urinary catheter
94	Insert a suprapubic catheter
82	Interpret intravenous pyelogram
76	Vasectomy
**Eyes**	
70	Subconjunctival injections
70	Use a Schiotz tonometer
100	Fundoscopy (diabetes, hypertension)
88	Instil drops or apply ointment
100	Remove foreign body from the eye
100	Test for squint
100	Washout of eyes (chemical burns)
**Ear, nose and throat**	
82	Assess hearing loss
82	Reduce a fractured nose
100	Remove a foreign body from ear and nose
100	Syringe, dry swab an ear
94	Take a throat swab
100	Manage epistaxis (cautery, packing)
82	Perform Rinne and Weber tests
100	Suture a pinna lobe
82	Drain a peritonsillar abscess
**Skin**	
82	Inject keloids
82	Phenol ablation of ingrown toenail
100	Excise sebaceous cyst (other lumps, bumps)
100	Apply a compression dressing to venous leg ulcer
100	Cryotherapy or cauterisation
100	Skin biopsy (punch and shave) or skin scrapes
100	Wide-needle aspiration biopsy lymph node
**Pregnancy**	
94	Obstetric ultrasound
100	Interpret antenatal growth chart
94	Assess foetal well-being during labour
100	Episiotomy and suturing
94	Examine a pregnant woman
94	Examine progress during labour and use partogram
94	Normal vaginal delivery
100	Speculum examination
100	Apply and interpret the cardiotocograph
94	Assess foetal movement
100	Assisted vaginal delivery or vacuum extraction or forceps
94	Caesarean section
100	Evacuation of uterus
100	Manual removal of placenta
94	Repair of third-degree tear
82	Pelvic ultrasound (transvaginal)
**Woman’s health**	
88	Culdocentesis
100	Hormone implants
82	Laparotomy for ectopic pregnancy
70	Termination of pregnancy
100	Insertion of intrauterine contraceptive device
100	Papanicolaou smears
88	Dilatation and curettage
88	Drainage of Bartholin’s abscess or cyst
76	Endometrial biopsy or sampling
94	Fine-needle aspiration biopsy of breast lump
88	Tubal ligation
71	Cervical polyp removal
**Newborn**	
100	Well newborn check
100	Assess gestational age at birth
100	Kangaroo mother care
100	Resuscitate a newborn
94	Umbilical vein catheterisation
**Consultation**	
100	Patient-centred consultation
100	Use genogram and eco-map
100	Develop and use flowcharts for chronic care
100	Motivate behaviour change
100	Assess and consult families, couples
100	Shared consultation to capacitate nurse practitioner
100	Counselling skills for HIV, termination of pregnancy, sexual assault
100	Break bad news
94	Mini–Mental State Examination
100	Use problem-orientated medical record
94	Conduct a family conference
100	Cope with language barriers
100	Holistic assessment and management
100	Sexual history and counselling
**Emergency**	
100	Calculate % burn
100	Manage choking
94	Give oxygen
100	Immobilise spine
100	Intubate and manage airway
94	Measure the Glasgow Coma Scale
94	Administer rabies prophylaxis
100	Advanced cardiopulmonary resuscitation – Adult
94	Advanced cardiopulmonary resuscitation – Child
100	Debride wounds or burns
100	Gastric lavage
100	Give a blood transfusion
100	Incision and drainage of abscesses
100	Insert chest drain
100	Insert nasogastric tube
100	Interpret radiographs in trauma
88	Intravenous cut down
94	Manage snake bite
100	Primary survey
100	Relieve tension pneumothorax
100	Remove a splinter fish hook
100	Secondary survey
88	Selecting emergency equipment for doctors’ bag or emergency tray
94	Suture lacerations
82	Transport critically ill
76	Cricothyroidotomy
70	Insert central line
**Orthopaedics**	
94	Apply finger and hand splints
94	Apply casts to upper and lower limb
100	Closed reductions for hand, forearm, tibia, fibula
88	Set up traction skeletal and skin
94	Reduce elbow dislocation
82	Reduce hip dislocation
94	Reduce radial head dislocation
100	Reduce shoulder dislocation
82	Excise ganglion
88	Amputations – fingers
76	Apply club foot cast
76	Debridement of open fractures
76	Fasciotomy
**Anaesthetics**	
94	Injections – intra-dermal, subcutaneous, intramuscular, deep intramuscular, sub-conjunctival
100	Ring block
94	Administer oxygen
88	Check Boyle’s machine
94	Control airways with mask
82	General anaesthetic
82	Inhalation induction
82	Intravenous induction
94	Intubate and ventilate patient
82	Ketamine anaesthesia
88	Monitor patient during anaesthetic
88	Recover patient – recovery room
82	Reverse muscle relaxation (mixed drugs)
88	Set airflow – Magill Circle, T-piece
94	Spinal anaesthetic
70	Sterilise equipment
94	Ventilate patient mask and hand
76	Biers block
82	Brachial block
**Child health**	
100	Assess growth and classify malnutrition
94	Capillary blood sampling – finger and heel
94	Chest radiograph in child
100	Developmental assessment
100	How to do and interpret Tine and Mantoux tests
94	Intraosseous line
100	Intravenous access in a child
94	Lumbar puncture in a child
100	Manage problems using the integrated management of childhood
94	Suprapubic bladder puncture
100	Venepuncture – upper limb and external jugular vein
94	Manage neonatal jaundice with phototherapy
**Clinical administration**	
100	Complete sick certificates
100	Complete death certificates
100	Certify patient under Mental Health Care Act
100	Making appropriate referrals and letters
100	Managing a clinic for chronic care, for example, HIV and ARVs
76	Perform work assessment and complete disability grant forms
**Forensic**	
100	Assess, manage and document drunken driving
100	Assess, manage and document interpersonal violence
100	Assess, manage and document sexual assault
100	Complete J-88 form following assault
**Palliative care**	
100	Counselling of a dying patient
70	Hypodermoclysis (subcutaneous infusion)
76	Set up a syringe driver
**Clinical governance**	
100	Able to contribute to the development or revision of guidelines
100	Able to facilitate the implementation of clinical guidelines within the subdistrict
100	Able to improve quality of care by facilitating quality improvement cycles (including the audit of clinical care as one step in the cycle)
100	Able to improve cost-effectiveness through reflection on routinely collected data, particularly rational prescribing and use of investigations
100	Build capability and quality care through teaching, training and mentoring
100	Able to critically appraise new evidence
76	Able to appraise the competence of new clinicians and set appropriate levels of independence versus support
94	Able to evaluate the quality of care in relation to the relevant clinically orientated national core standards
**Community-orientated primary care**	
94	Able to do a home visit
100	Able to make a community diagnosis, and interpret and prioritise health indicators
100	Able to promote health in communities
**Teaching and training**	
100	Able to plan and implement a teaching or continuing professional development activity
100	Able to use a portfolio of learning
100	Able to mentor a colleague
100	Able to facilitate small group learning
100	Able to prepare and give a presentation

**TABLE 2:** Skills not needed in the training of family physicians (*N* = 17).

Consensus score (%)	Clinical skills
**Adults – abdomen**	
76	Anal dilatation
**Adults – urology**	
88	El-Ghorab shunt for priapism
**Eyes**	
82	Subjective refraction and dispense ‘stock’ glasses

**TABLE 3:** Optional or elective skills that are required in certain limited settings such as rural or remote district hospitals.

Consensus score (%)	Clinical skills
**Perform common side-room tests**	
76	Microscopy of vaginal discharge (wet mount, potassium hydroxide)
**Adult health – general**	
76	Bone marrow puncture technique and smear
76	Microscopy of cerebrospinal fluid
94	Thin and thick smears for malaria
**Adults – abdomen**	
76	Abdominal ultrasound
70	Anal sphincterotomy
88	Gastroscopy
76	*Helicobacter pylori* testing
76	Peritoneal dialysis
88	Repair a hernia
88	Sigmoidoscopy
76	Liver biopsy
**Adults – chest**	
76	Echocardiogram
**Adults – urology**	
76	Hydrocoelectomy
82	Bilateral capsular orchidectomy
76	Cystoscopy
76	Prostate biopsy
**Eyes**	
82	Slit-lamp examination
**Ear, nose and throat**	
82	Indirect laryngoscopy
**Woman’s health**	
76	Cone biopsy of cervix
71	Colposcopy
76	Hysterectomy
76	Large loop excision of the transformation zone for cervix
**Orthopaedics**	
70	Open reductions – pins and screws
**Child health**	
71	Extradural tap
**Dental**	
76	Dental extraction
70	Wiring of teeth for mandibular fracture
**Forensic**	
82	Medico legal post-mortem

**TABLE 4:** Skills for which no consensus could be reached.

Clinical skills	Retain (%)	Delete (%)	Elective (%)
**Perform common side-room tests**			
Microscopy of urine	47		53
**Adult health – general**			
Doppler ultrasound – For peripheral vascular disease	30	6	64
**Adults – abdomen**			
Appendicectomy	53	6	41
Injection of haemorrhoids	41		59
Rubber-banding of haemorrhoids	35	6	59
**Adults – chest**			
Pleural biopsy	65		35
**Ear, nose and throat**			
Tonsillectomy or adenoidectomy	41	12	47
**Skin**			
Skin patch testing	24	12	64
**Pregnancy**			
Clinical pelvimetry	47	35	18
Amniocentesis	18	24	58
**Anaesthetics**			
Epidural	53	6	41

## Discussion

The skills list generated by this study represents a new national consensus on the clinical skill outcomes for postgraduate family medicine training programmes in South Africa. Consensus was reached on 242 core clinical skills, from which the panel decided that 211 should be retained, 3 should be deleted and 28 should be made elective. The panel could not reach consensus on 11 skills in the final questionnaire.

In a previous study, consensus was obtained on 168 core clinical skills that should be performed independently at the end of postgraduate training in South Africa.^4^ This study therefore reflects how family medicine training has expanded since the previous study was conducted in 2008 as an additional 43 skills were added to the new list. Many of the new skills are derived from additional roles that have been included in the list such as leadership of clinical governance, champion of community-orientated primary care and capacity building of the health care team through skills in teaching and learning. Previously, a more narrow definition of clinical skills was utilised, but in this study the panel felt these additional roles and their associated skills should be included.

While the 211 skills that were retained represent the core skills that every training programme should focus on, the list of elective skills is more open ended. The panel reached consensus on 28 skills that would typically be regarded as useful in some settings and would need to be mastered if the FP worked there. This list is however not exhaustive and other skills could be regarded as elective if the circumstances required them. Nevertheless, the 28 elective skills give some guidance to the training programmes on additional skills that may be needed.

The underlying assumption in the training programmes is that FPs should be prepared to work across the district hospital–primary care dyad. Often, the FP moves between both these settings and, for example, may be on call in the district hospital and providing outreach and support to primary care. In some settings, particularly metropolitan areas, the FP may only work in primary care and the list of required skills will be reduced. Nevertheless, every FP on qualifying should be competent to work across the whole district health system.

One way in which training programmes might decide on operationalising the elective list is by individualising each registrar’s future practice intent. For example, those intending to work in remote rural areas may require additional skills. Provinces may also have specific expectations of the skill set required of FPs.

In countries such as Canada and Australia, family medicine residency programmes offer a one- to two-year advanced procedural training course upon completion of family medicine training.^14,15^ In Australia, training for ambulatory general practice and for rural medicine are separated, whereas in South Africa these two have been combined into one training programme. In South Africa and India, the need for FPs to have an extended range of skills in anaesthesia, obstetrics and surgery is clear. This may create a tension between training FPs to offer these more procedural skills, for example at the district hospital, where such skills are often missing, versus training them to be primarily part of the primary care team, as in Brazil or China.^16^

A similar study conducted in Canada only identified 65 core clinical skills and 15 enhanced skills and emphasised that physicians who worked in more rural areas were more likely to perform more of the core clinical skills as opposed to their counterparts in urban settings.^14^ A more recent study in Australia produced a list of 112 procedural skills that should be taught in general practice vocational training.^15^ No comparable lists were found for clinical skill outcomes or procedural skills for family medicine residencies in China, India, Brazil or other African countries.

In South Africa, there is still ambivalence amongst policymakers about the role of the FP, and we hope that experience with current FPs and the national position paper as well as ongoing research will help to establish consensus on the contribution of the FP and the need for a trained expert generalist as part of the system.^13^

### Limitations

Only 17 of the 36 invited panel members agreed to participate in the study; therefore, this study was more limited in its representation of experts than intended. There were no representatives from the Universities of Cape Town and Limpopo and none from the provinces of North West, Northern Cape, Limpopo and Mpumalanga. However, the health needs of the provinces that were included are likely to be similar to those omitted and included both urban and rural populations. The two training programmes were also unlikely to have widely differing views to their colleagues. Overall, there was a balance of different categories of experts although fewer than intended in each category. No specialists in surgery and orthopaedics were included.

### Recommendations

The findings of this study will be considered by the National Education and Training Committee of the South African Academy of FPs in order to approve changes to the national learning outcomes and to decide how to handle the skills on which no consensus could be reached. Out of the 11 skills on which no consensus was reached, the panel agreed (> 70%) that 10 of them should not be deleted and therefore they should be considered for inclusion as at least elective skills. The panel had no consensus on retaining clinical pelvimetry as a skill, and it may be necessary to review the evidence in order to make a decision. It is important that the consensus of the panel be consistent with the latest evidence on the effectiveness of clinical skills, particularly when no consensus was reached. The recommendations of the Academy will also be presented to the College of Family Physicians to ensure that the national examination takes the new consensus into account.

Training programmes should then ensure that all registrars have the opportunity to become competent in these skills and that their learning is adequately recorded in the portfolio of learning, which will also need to be adapted. Clinical trainers must also be able to create a conducive learning environment within which they can observe clinical skills and give skilful feedback. The Academy is pioneering a short course for the training of clinical trainers and a process of quality assurance to raise the standard of clinical training across all programmes.

Policymakers and managers who are responsible for the district health services as well as health care providers and funders in the private sector should take cognisance of the clinical skills set defined in this study for the training of FPs. This may help them in understanding the roles and competencies of the FP and their contribution within the health care system.

## Conclusion

This study defined a new set of clinical skills for the training of FPs in South Africa which consists of 211 core skills and 28 elective skills. Consensus could not be reached on 11 other clinical skills. The professional bodies for the discipline of family medicine must revise the national learning outcomes and national examination and training programmes to be aligned with this new consensus on the required clinical skills.
